# Relationships of Haptoglobin Phenotypes with Systemic Inflammation and the Severity of Chronic Obstructive Pulmonary Disease

**DOI:** 10.1038/s41598-018-37406-9

**Published:** 2019-01-17

**Authors:** Pao-Lin Lee, Kang-Yun Lee, Tsai-Mu Cheng, Hsiao-Chi Chuang, Sheng-Ming Wu, Po-Hao Feng, Wen-Te Liu, Kuan-Yuan Chen, Shu-Chuan Ho

**Affiliations:** 1 Department of Respiratory Therapy, Cardinal Tien Hospital, New Taipei City, Taiwan; 2 Division of Thoracic Medicine, Department of Internal Medicine, Shuang Ho Hospital, Taipei Medical University, Taipei, Taiwan; 3 Division of Thoracic Medicine, School of Medicine, College of Medicine, Taipei Medical University, Taipei, Taiwan; 4 Institute for Translational Medicine, College of Medical Science and Technology, Taipei Medical University, Taipei, Taiwan; 5 School of Respiratory Therapy, College of Medicine, Taipei Medical University, Taipei, Taiwan

## Abstract

Chronic obstructive pulmonary disease (COPD) is caused by chronic inflammation. Many inflammatory mediators induce the low grade systemic inflammation of COPD. Haptoglobin (Hp) is synthesized in the liver and by lung epithelial and alveolar macrophage cells. However, associations of the serum concentration and phenotype of Hp with COPD are unclear. Therefore, we explored the association of the Hp concentration and Hp phenotype with the inflammatory response and COPD disease severity. We included healthy subjects and COPD patients. The Hp phenotype was categorized by SDS native-PAGE, and concentrations were determined by ELISA. In this trial Hp concentrations in COPD groups were significantly higher than those in healthy controls. There was a significant negative correlation between the Hp concentration and FEV_1_(%) (*p* < 0.001), while IL-6 and 8-isoprostane were positively correlated with the Hp concentration. As to the Hp phenotype, there were significant negative correlations between the FEV_1_ and both Hp2-1 and Hp2-2; IL-6 and 8-isoprostane were significantly positively correlated with Hp2-1 and Hp2-2. The ROC curve analysis of the Hp concentration was significantly higher than CRP. Hp concentrations and phenotype were positively correlated with the severity of COPD, especially Hp2-2. In the future, Hp can be considered a novel biomarker for identifying COPD.

## Introduction

Chronic obstructive pulmonary disease (COPD) is characterized by a progressive airflow limitation that is not fully reversible^1^. COPD is a highly complex inflammatory disease involving many cytokines and mediators, such that blocking a single cytokine does not have a clinically significant effect^2^, in the presence of systemic inflammation, including C-reactive protein (CRP), interleukin (IL)-6, fibrinogen, activated leukocytes, and tumor necrosis factor (TNF)-α^3^. Systemic inflammation indicates an increase in plasma levels of various inflammatory proteins and acute phase reactants belonging to different biological pathways in COPD, which were significantly associated with the disease severity^4^.

Haptoglobin (Hp) is a selective suppressor of certain monocyte functions and may thus be considered a model protein for studying the anti-inflammatory potential of acute-phase proteins^5^. Hp originates from the liver, recently studies found that the plasma Hp is synthesized and expressed in the human lung^6^, pulmonary haptoglobin (pHp) is part of the surfactant system^7^, a native function of pHp and CD163 are immunoregulatory elements due to local expression, regulation and secretion during lung infection and inflammatory immune response of the respiratory system^8^, IL-6 and dexamethsone (DEX) are capable of upregulating the synthesis of Hp^8^.

It responds to the release of cytokines such as IL-6, IL-1, and TNF-α by the human liver, and by lung epithelial cells and alveolar macrophages, and increases in inflammation and infections^5,9^. The serum concentration of this protein is usually lower than normal in tissues. Changes in serum levels of this protein in asthmatic patients after allergen skin test trials were reported in children^10^.

The lungs are a major site of the extrahepatic synthesis of Hp. This protein protects the lungs against inflammatory agents, and its presence prevents lung injury^11,12^. Its deficiency leads to lung tissue destruction caused by natural bacteriostatic neutrophil function, leading to initiation of emphysema and COPD. A protein deficiency leads to early-onset emphysema, and usually the incidence of disease is exacerbated by smoking and in some cases progresses to asthma^13^. This protein exists in humans as three main phenotypes of Hp1-1, Hp2-2, and Hp2-1, which are determined by two alleles: Hp1 and Hp2^14^. Hp2-2 is a weaker antioxidant than Hp1-1 and Hp2-1^15^. There are functional differences between different Hp phenotypes. In this study, we investigated relationships of Hp phenotypes with systemic inflammation and disease severity in patients with COPD.

## Results

**Figure 1:**
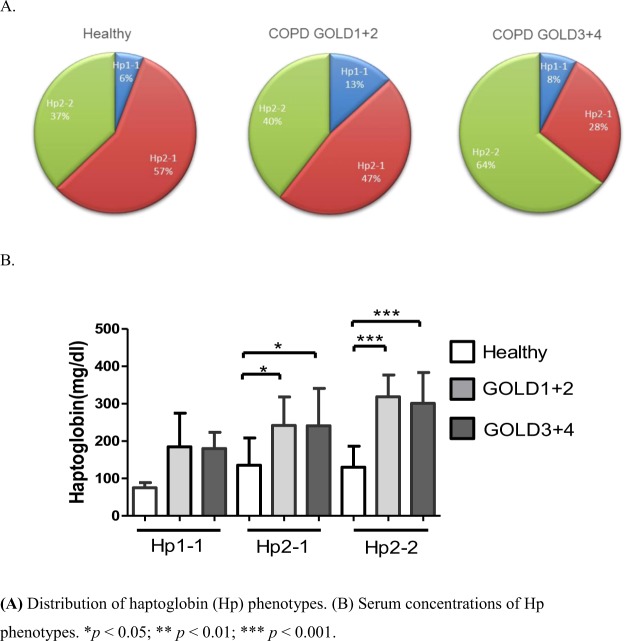
(**A**) Distribution of haptoglobin (Hp) phenotypes in healthy subjects and in chronic obstructive pulmonary disease (COPD) GOLD 1 + 2 and GOLD 3 + 4 patients. (**B**) Serum concentrations of Hp phenotypes in healthy subjects and COPD GOLD 1 + 2 and GOLD 3 + 4 patients. *p < 0.05; **p < 0.01; ***p < 0.001.

**Figure 2:**
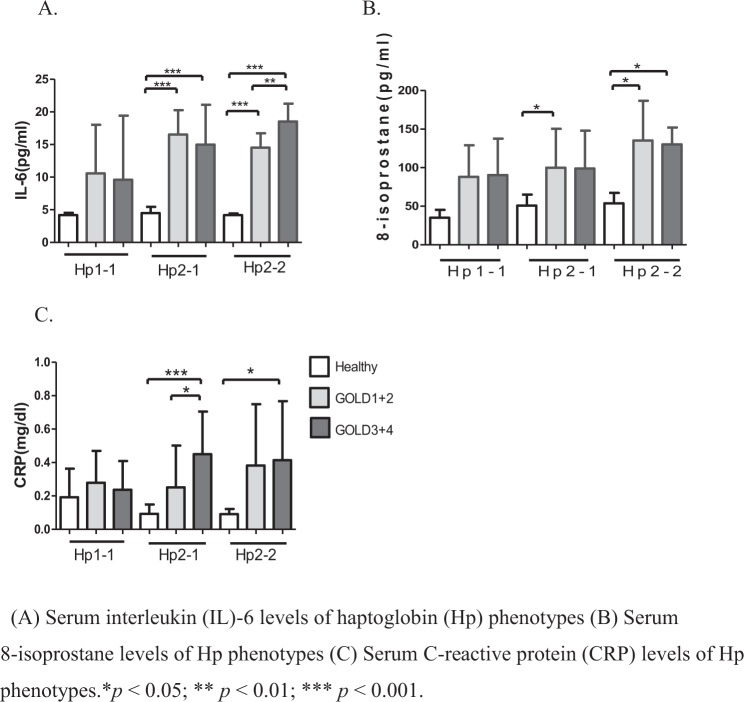
(**A**) Serum interleukin (IL)-6 levels of haptoglobin (Hp) phenotypes in healthy subjects and in chronic obstructive pulmonary disease (COPD) GOLD 1 + 2 and GOLD 3 + 4 patients. (**B**) Serum 8-isoprostane levels of Hp phenotypes in healthy subjects and COPD GOLD 1 + 2 and GOLD 3 + 4 patients. (**C**) Serum C-reactive protein (CRP) levels of Hp phenotypes in healthy subjects and COPD GOLD 1 + 2 and GOLD 3 + 4 patients. *p < 0.05; **p < 0.01; ***p < 0.001.

In total, 126 participants, 35 in the control group and 91 in the COPD groups (38 COPD GOLD 1 + 2 group and 53 COPD GOLD3 + 4 group), were included in the study. Table 1 summarizes baseline characteristics of these participants. Several baseline characteristics demonstrated significant between-group differences. A significantly higher proportion of participants in the COPD group were older, men, smokers, steroid treatment, had lower pulmonary function tests compared to participants in the control group (all *p* < 0.05). Serum concentrations and phenotype levels of Hp were significantly higher in the COPD group compared to the control group (all *p* < 0.05). There was a significant difference in the proportion of participants with different Hp phenotypes, and a much higher proportion of COPD groups with high Hp2-1 and Hp2-2, but not Hp1-1 (Fig. 1A), concentrations was found compared to the healthy group(Fig. 1B). As to systemic inflammatory cytokines, we found that IL-6, 8-isoprostane, and CRP concentrations were significantly higher in the COPD groups compared to the control group (all *p* < 0.05, Fig. 2), which shows that systemic inflammatory cytokines were related to higher Hp2-1 and Hp2-2 levels in the COPD groups than in the healthy control group, but the Hp1-1 phenotype did not significantly differ. Table 2 shows correlations of baseline characteristics of FEV_1_, IL-6, and 8-isoprostane serum levels with phenotype concentrations of Hp. The serum concentration of Hp was significantly negatively correlated with gender and FEV_1_ (both *p* < 0.01), and was positively correlated with IL-6 and 8-isoprostane (both *p* < 0.01). In the three Hp phenotype groups, in participants with a high Hp1-1 concentration, no significant correlations were detected among all variables. The Hp2-1 concentration was significantly negatively correlated with FEV_1_ (*p* < 0.05) and was positively correlated with IL-6 and 8-isoprostane (both *p* < 0.01). The Hp2-2 concentration was significantly negatively correlated with gender and FEV_1_ (both *p* < 0.05), and was positively correlated with age and IL-6 (both *p* < 0.05). The other results showed that BMI, steroid treatment and CRP were not significantly correlated with total Hp concentration and three Hp phenotypes.

**Table 1:** Characteristics of study subjects.

Indicators	Healthy (n = 35)	COPD GOLD1 + 2 (n = 38)	COPD GOLD3 + 4 (n = 53)	P-value
**Age**, **yr**	63.9 ± 6.5	68.2 ± 8.8a	68.51 ± 6.8a	0.011
**Male**, **%**	12 (34.3)	36 (94.7)	50 (94.3)	<0.001^#^
**BMI**, **kg/m**^**2**^	23.9 ± 3.3	23.6 ± 3.6	24 ± 4.3	0.874
**Smoke status**				<0.001^#^
Smoking, N (%)	5 (14.3)	9 (23.7)	15 (28.3)	
Never smoke, N (%)	27 (77.1)	7 (18.4)	10 (18.9)	
Ex-smoking, N (%)	3 (8.6)	22 (57.9)	28 (52.8)	
**Steroid treatment**				<0.001^#^
Without, N (%)	35 (100)	16 (42.1)	14 (26.4)	
With, N (%)^@^	0 (0)	22 (57.9)	39 (73.6)	
**Pulmonary Function Tests**				
FEV_1_, %predicted	92.8 ± 14.7	60.1 ± 8.5^a^	34.28 ± 9.5^a,b^	<0.001
FVC, %predicted	90.3 ± 15.3	79.3 ± 13.5^a^	57.7 ± 14.4^a,b^	<0.001
FEV_1_/FVC, %predicted	83.0 ± 5.0	59.7 ± 8.3^a^	47.4 ± 9.8^a,b^	<0.001
**Haptoglobin**, **mg/dl**	126.8 ± 63.0	262.3 ± 84.7^a^	273.29 ± 93^a^	<0.001
**Haptoglobin phenotype**				0.038
Hp1-1, mg/dl	75.0 ± 14.1	184.8 ± 90.0	180.0 ± 35.0	0.199
Hp2-1, mg/dl	135.4 ± 73.3	242.0 ± 76.0^a^	240.9 ± 100.2^a^	<0.050
Hp2-2, mg/dl	130.5 ± 56.3	318.5 ± 58.5^c^	300.9 ± 82.7^c^	<0.001
**IL-6**, **pg/ml**	4.38 ± 0.8	14.68 ± 4.8^a^	15.79 ± 6.1^a^	<0.001
**CRP**, **mg/dl**	0.1 ± 0.07	0.31 ± 0.32^a^	0.58 ± 0.7^a^	<0.001
**8-isoprostane**, **pg/ml**	50.5 ± 14.2	114.3 ± 51.3^a^	120.9 ± 78.6^a^	<0.001

Notes: Data are presented as % or mean ± SD; ^a^P < 0.05 versus Healthy control; ^b^P < 0.05 versus GOLD1 + 2; ^c^P < 0.001 versus Healthy control;

^#^Analysis of chi-square test; ^@^inhaled steroid COPD GOLD1 + 2 n = 22, COPD GOLD3 + 4 n = 35, systemic steroid COPD GOLD1 + 2 n = 0, COPD.

GOLD3 + 4 n = 4; Abbreviations: BMI, Body mass index; COPD, Chronic Obstructive Pulmonary Disease; GOLD, Global initiative for Chronic.

Obstructive Lung Disease; FEV_1_, Forced expiratory volume in first second; FVC, Forced vital capacity; IL-6, Interleukin-6; CRP, C-Reactive Protein.

**Table 2:** Spearman’s correlation coefficients for total haptoglobin and haptoglobin phenotype concentrations and systemic inflammatory cytokines.

Variables	Total haptoglobin	Haptoglobin phenotype		
		Hp1-1	Hp2-1	Hp2-2
Age, years	0.076	−0.293	−0.157	0.353*
Gender^a^	−0.378**	−0.523	−0.109	−0.632**
BMI, kg/m^2^	−0.004	0.097	0.161	−0.074
Smoke status	−0.16	0.582	−0.171	−0.182
Steroid treatment	0.086	0.289	0.154	0.027
FEV_1_, %	−0.499	−0.559	−0.347*	−0.555***
IL-6, pg/ml^a^	0.671**	0.435	0.570**	0.786**
CRP, mg/dl^b^	0.148	−0.182	0.166	0.139
8-isoprostane, pg/ml^c^	0.389**	0.648	0.560*	0.21

Notes: ^a^n = 63; ^b^n = 97; ^c^n = 56;

Abbreviations: BMI, Body mass index; FEV_1_, Force expiratory volume in first second; IL-6, Interleukin-6;

CRP, C-Reactive Protein; Hp, haptoglobin.

Table 3 shows the multiple linear regression analysis of the three Hp phenotypes with gender, smoking status, FEV_1_, IL-6, and 8-isoprostane. We adjusted for all variables that could possibly influence the concentration of Hp (i.e., age, gender, smoking status, the body-mass index, and FEV_1_). After adjusting for all of those variables, the presence of Hp phenotypes still significantly differed between healthy and COPD participants. Results showed that gender, FEV_1_, IL-6, and 8-isoprostane were independent factors for different Hp phenotypes. Gender was negatively associated with the Hp2-2 concentration (*p* < 0.01) (Hp phenotype levels were adjusted for age, gender, and smoking status). A low FEV_1_ was associated with high concentrations of the three Hp phenotypes (all *p* < 0.05). High IL-6 was associated with high concentrations of the three Hp phenotypes (all *p* < 0.05). High 8-isoprostane was associated with high Hp1-1 and Hp2-1 concentrations (both *p* < 0.05), but was not associated with Hp2-2.

**Table 3:** Linear regression analysis of haptoglobin phenotypes in the participants.

Variables	HP1-1			HP2-1			HP2-2		
	B	t	p	B	t	p	B	t	p
Gender	−71.61	−1.24	0.245	−33.48	−0.8	0.428	−155.7	−6.22	<0.001
Smoke status	−49.93	0.52	1.54	−37.58	−1.46	0.152	−21.29	−1.35	0.182
FEV1, %	−2.35	−2.93	0.026	−1.54	−2.46	0.019	−1.89	−1.1	<0.001
IL-6, pg/ml	7.25	2.9	0.027	8.41	3.61	0.002	11.93	4.8	<0.001
8-isoprostane, pg/ml	2.08	19.62	0.032	1.29	2.37	0.032	0.1	0.39	0.708

Abbreviations: FEV_1_, Forced expiratory volume in first second; IL-6, interleukin-6; CRP, C-Reactive Protein.

**Figure 3:**
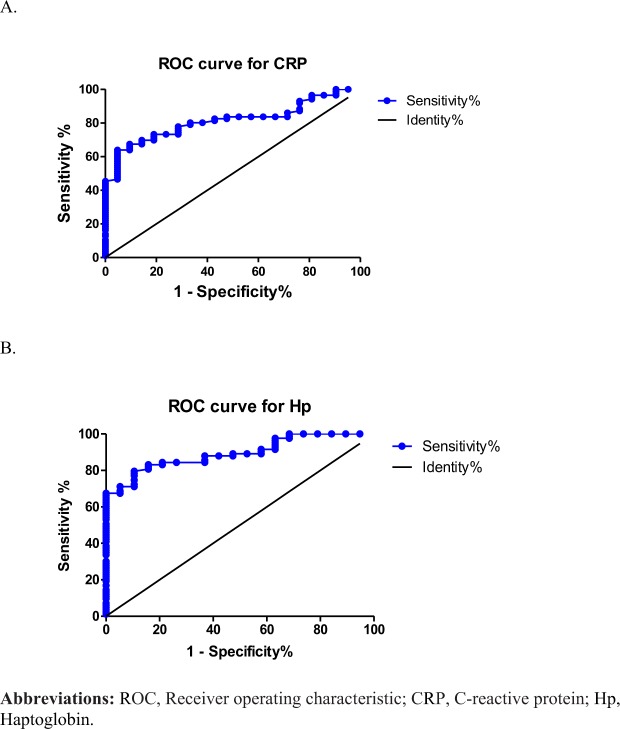
Performance of serum C-reactive protein (CRP) and haptoglobin (Hp) in receiver operating characteristic curve analyses between healthy subjects and chronic obstructive pulmonary disease (COPD) patients.

**Figure 4:**
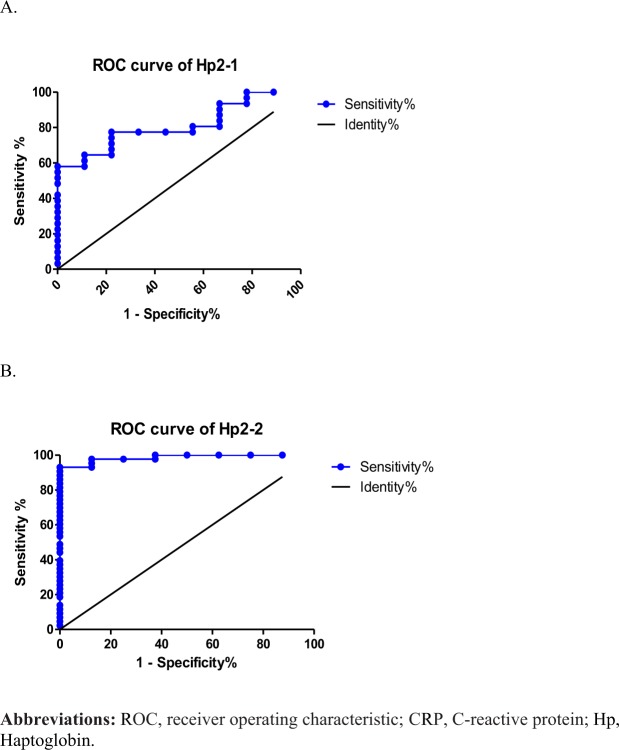
Performance of serum haptoglobin 2-1 (Hp2-1) and Hp2-2 phenotypes in receiver operating characteristic curve analyses between healthy subjects and chronic obstructive pulmonary disease (COPD) patients.

Figure 3 shows the ROC curve of sensitivity and specificity of CRP and serum levels of Hp for discriminating between the healthy and COPD groups. The area under the ROC curve (AUC) was 0.8131 (0.731~0.896, *p* < 0.01) for CRP concentrations in participants (Fig. 3A). The AUC was 0.8938 (0.831~0.957, *p* < 0.01) for serum levels of Hp in participants (Fig. 3B). The serum Hp cutoff value determined by the Youden index for participants was 187.0 mg/dl. The population of participants with Hp1-1 was too small to conclude whether it can be used to discriminate between healthy and COPD phenotypes. Further, the ROC curve of Hp2-1 and Hp2-2 levels for discriminating between the healthy and COPD groups. The AUC was 0.810 (0.674~0.946, *p* < 0.01) for the Hp2-1 concentration in participants (Fig. 4A).The AUC was 0.985 (0.959~1.000, *p* < 0.001) for the Hp2-2 level in participants (Fig. 4B). The Hp2-2 cutoff value determined by the Youden index for participants was 192 mg/dl.

## Discussion

This study showed four major results: (1) distributions of Hp phenotypes differed between healthy participants and COPD patients; (2) concentrations of Hp phenotypes were positively significantly related to systemic inflammation and oxidative stress in COPD patients; (3) concentrations of Hp phenotypes were negatively related to the FEV_1_ in participants; and (4) serum concentrations of Hp phenotypes were significantly higher in COPD patients than in healthy participants.

As to distributions of Hp phenotypes among various populations in the world^15^, the majority of Asians have very low HP^1F^ values compared to those on other continents, with Indians averaging about 0.05, with its virtual absence in Japanese, Koreans, and Taiwanese^16^. The distribution of the Hp1 allele is higher in Blacks than in Caucasians and even more so than in populations of Southeast Asia^15^. There was a decreased Hp2-1 frequency in those with a family history of bronchial asthma, and a low Hp2-2 frequency in those with adenocarcinoma of the lungs^15^. Different Hp phenotypes exhibited equal susceptibilities to pulmonary tuberculosis^17^. Tuberculosis patients with the Hp2-2 phenotype had a higher risk of mortality^17^. As to functional differences between Hp phenotypes, Hp2-2 may provide less protection against hemoglobin iron-driven peroxidation leading to lower concentrations of ascorbic acid compared to those carrying Hp1-1 and Hp2-1^18,19^. This study found a high Hp2-2 frequency in the most severe COPD GOLD3 + 4 group, and particularly Hp2-2 phenotype concentrations may be most capable of predicting systemic inflammation, according to the ROC analysis. Therefore, Hp may be used to differentiate severity in COPD patients, and it can be used to identify COPD severity.

Hp is an acute-phase protein, and its plasma concentration increases in response to a variety of stimuli^5^. A past study noted that plasma Hp increased in patients with an abdominal aortic aneurysm^20^ and coronary artery disease (CAD)^21^. Hp may also play a proatherogenic role in which Hp acts as a chemoattractant to pre-B lymphocytes and monocytes in inflammatory adipose tissue^22^. Circulating concentrations of Hp were found to increase in parallel with COPD severity in patients^4^. Our study showed that the serum Hp concentration significantly increased and was positively related to systemic inflammation and oxidative stress in COPD patients, and this indicator significantly predicted systemic inflammation and oxidative stress, but not significantly correlated with steroid treatment for total Hp and Hp phenotype concentration.

Total Hp concentrations vary with phenotype^23^, in Chinese subjects, Hp2-2 concentrations were highest in males^19^, and our study had similar results. An increase in Hp levels may generate a feedback dampening of the severity of cytokine release and may protect against endotoxin-induced effects^5,24^. As a nitric oxide (NO) scavenger the Hp-Hb complex has a role in regulating NO bioavailability and vascular homeostasis; Hp inhibits prostaglandin synthesis and so has an anti-inflammatory action^25^. This study found that Hp concentrations were significantly positively correlated with IL-6 and 8-isoprostane (both *p* < 0.01); IL-6 was associated with all three Hp phenotype concentrations (all *p* < 0.05). 8-Isoprostane was associated with subtype Hp1-1 and Hp2-1 concentrations (both *p* < 0.05), but was not associated with Hp2-2.

Our study has several limitations. (1) This study was relatively small in size, and study participants were recruited from a single hospital. Further, larger sample sizes from different centers are needed to confirm our results. (2) Furthermore, several differences in baseline characteristics also existed among study participants in the three groups. These cohort characteristics and differences may have introduced bias and affected the results of our study to some extent. Most of the COPD patients referred to this center were male; hence, we could not assess the effect of gender. Further samples are needed to increase female participation. (3) The severity of COPD was evaluated by FEV_1_ alone. However, we know that the GOLD evaluation of COPD severity can be more complex and comprehensive. (4) Many methods can be used to identify Hp phenotypes, but we only used electrophoretic techniques. In the future, polymerase chain reaction-based methods may be developed to enable the identification of Hp allele types in subjects.

## Conclusions

Hp concentrations were positively correlated with the severity of COPD, and IL-6 and 8-isoprostane serum concentrations. The Hp phenotype also influenced COPD severity and IL-6, especially the Hp2-2 phenotype, for which the disease severity and IL-6 levels were significantly higher than those of Hp1-1 and Hp2-1. In addition, the area under ROC curve of Hp concentration was significantly higher than that of CRP. In the future, Hp may be considered a novel biomarker for identifying COPD severity.

## Methods

### Ethics

The Ethics Committee of Taipei Medical University-Joint Institutional Review Board (Taipei, Taiwan) approved the study protocol. All subjects received written and oral information prior to inclusion and provided written informed consent. All methods were performed in accordance with the relevant guidelines and regulations.

### Study participants

All study participants provided written consent. We enrolled 91 patients with COPD and 35 subjects as healthy controls from Shuang Ho Hospital (New Taipei City, Taiwan) between March 2014 and April 2016. Patients with COPD received a diagnosis from a physician and exhibited a post-bronchodilator forced expiratory volume in the first second (FEV_1_)/forced vital capacity (FVC) ratio of ≤70%. The classification of COPD severity followed the GOLD guidelines^26^. Healthy control subjects exhibited an FEV_1_/FVC ratio of ≥75% and an FEV_1_ of ≥80% of the predicted value. All subjects were aged 40~80 years at the time of inclusion. Subjects with a known malignant tumor, active inflammatory disease (such as asthma, bronchiectasis, or other non-COPD-related disease), or exacerbation during the 3 months prior to the study were not included.

### Hp phenotyping

The phenotypes of serum samples were determined by Hb-supplemented 6% native polyacrylamide gel electrophoresis (PAGE) and peroxidase chromogenic staining to detect Hb peroxidase activity, as described by Cheng *et al*. (Supplementary Fig. S1)^14^. Briefly, a sample containing 7 μL of serum was premixed with 5 μL of 8 mg/mL hemoglobin and equilibrated with 3 μL of sample buffer (0.625 mol/L Tris-base at pH 6.8, 50% glycerol, and 0.125 mg/L bromophenol blue). The mixture was run on a 6% native polyacrylamide gel (pH 8.8), with 4% polyacrylamide used as a top stacking gel (pH 6.8). Electrophoresis was performed at an initial voltage of 120 V, which was increased to 150 V when the dye front reached the separating gel. After electrophoresis, the Hp- was visualized by shaking the gel in freshly prepared peroxidase substrate (0.05% 3,3′-diaminobenzidine and 0.07% hydrogen peroxide in phosphate-buffered saline;). Results were confirmed by Western blotting using an α-chain-specific monoclonal antibody (mAb)^27^.

### Hp purification

Serum of each Hp phenotype was chromatographed on an mAb-based affinity column followed by a high-performance liquid chromatographic (HPLC) procedure as described by Cheng *et al*.^27^.

### Measurement of the human free Hp serum level

Human free serum concentrations of Hp were measured using a phenotype-matched standard sandwich enzyme-linked immunosorbent assay (ELISA) as described in a previous publication^28^.

### ELISA for IL-6, CRP, and 8-isoprostane

We used ELISAs to determine serum levels of IL-6, CRP (R&D Systems, Minneapolis, MN, USA), and 8-isoprostane (Cayman Chemical, Ann Arbor, MI, USA) according to manufacturer’s instructions.

### Statistical analysis

Patients’ demographic and clinical characteristics were summarized as the mean ± standard deviation for continuous data and the number (percent) for categorical data. For comparisons among multiple values, a one-way analysis of variance (ANOVA) with Turkey’s post-hoc test was used. A Pearson correlation analysis was performed to identify correlations of Hp concentrations with age, anthropometric indicators, and CRP, IL-6, and 8-isoporstane concentrations. A linear regression analysis was performed to identify associations between Hp phenotypes and participants’ characteristics. The optimal serum Hp concentration cutoff point for predicting COPD was determined based on maximization of the Youden index using a receiver operating characteristic (ROC) curve analysis. All statistical assessments were considered significant at *p* < 0.05. Statistical analyses were performed using SPSS 20.0 statistics software (SPSS, Chicago, IL, USA) and Graphpad Prism 5 software (GraphPad Software, San Diego, CA, USA).

## Supplementary information

Supplementary Information
