# Supplementary material for: Relationships of Haptoglobin Phenotypes with Systemic Inflammation and the Severity of Chronic Obstructive Pulmonary Disease
Source: Sci Rep. 2019 Jan 17;9:189. doi: 10.1038/s41598-018-37406-9 (PMC6336792; doi:10.1038/s41598-018-37406-9)
Supplement: Supplementary file 1 — Supplementary Information [file 41598_2018_37406_MOESM1_ESM.pdf]

Relationships of Haptoglobin Phenotypes with Systemic Inflammation and the Severity of Chronic Obstructive Pulmonary Disease.

(Supplementary Materials)

PaoLin Lee[1,5*],KangYun Lee[2,3*],TsaiMu Cheng[4],HsiaoChi Chuang[2,3,5],ShengMing Wu[2,3],PoHao Feng[2,3],WenTe Liu[2,3], KuanYuan Chen[2,3], ShuChuan Ho[2,5]

Affiliations:

1. Department of Respiratory Therapy, Cardinal Tien Hospital, New Taipei City, Taiwan.
2. Division of Thoracic Medicine, Department of Internal Medicine, Shuang Ho Hospital, Taipei Medical University, Taipei, Taiwan.
3. Division of Thoracic Medicine, School of Medicine, College of Medicine, Taipei Medical University, Taipei, Taiwan.
4. Institute for Translational Medicine, College of Medical Science and Technology, Taipei Medical University, Taipei, Taiwan.
5. School of Respiratory Therapy, College of Medicine, Taipei Medical University, Taipei, Taiwan.

*These authors contributed equally to this work

Corresponding author

ShuCuan Ho

School of Respiratory Therapy, College of Medicine, Taipei Medical University, 250 Wuxing Street, Taipei 11031, Taiwan.

Tel: +886-2-2736-1661 ext. 3512, Fax: +886-2-2739-1143

E-mail: shu-chuan@tmu.edu.tw

(A).

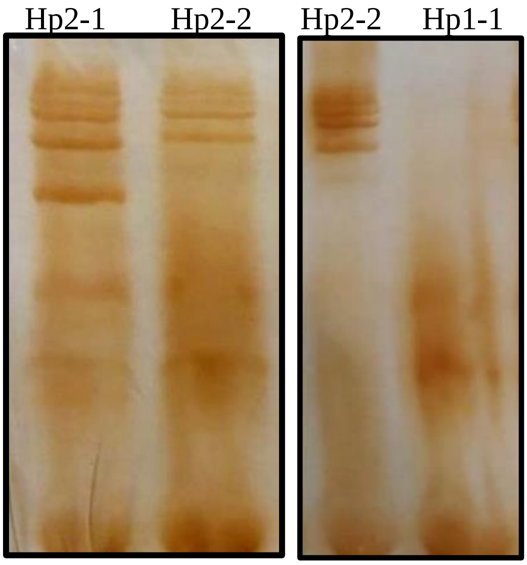

(B).

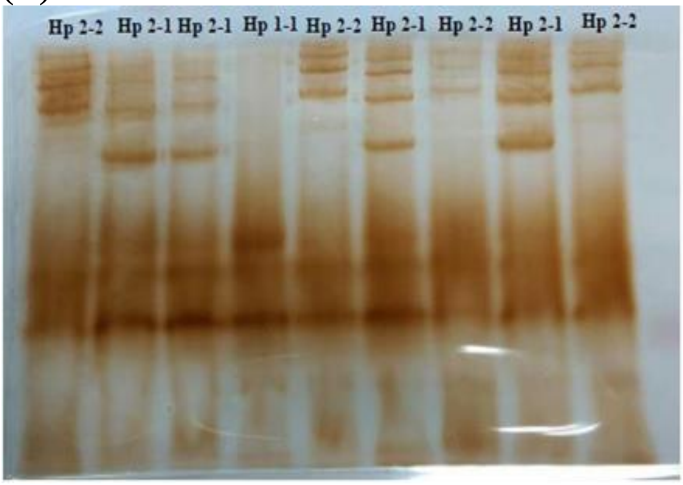

(C).

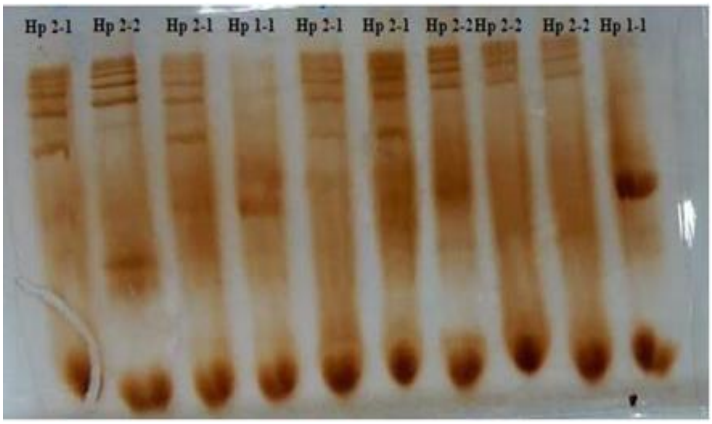

Supplementary Fig.1 (A) Hemoglobin-binding patterns of human plasma Hp on 6% native-PAGE. Lane 1, 2, 3 and 4, human plasma of Hp2-1, 2-2, 2-2 and 1-1 phenotypes with hemoglobin, respectively. (B) 6% native-PAGE in healthy subjects. (C) 6% native-PAGE in COPD patients.
